# Supplementary material for: The Reaction Mechanism of Loganic Acid Methyltransferase: A Molecular Dynamics Simulation and Quantum Mechanics Study
Source: Molecules. 2023 Jul 30;28(15):5767. doi: 10.3390/molecules28155767 (PMC10420828; doi:10.3390/molecules28155767)
Supplement: Supplementary file 1 [file molecules-28-05767-s001.zip › molecules-2530656-supplementary.pdf]

Supporting Information

The Reaction Mechanism of Loganic Acid Methyltransferase: A Molecular Dynamics Simulation and Quantum Mechanics Study

Mateusz Jędrzejewski, Łukasz Szeleszczuk and Dariusz Maciej Pisklak *

Department of Organic and Physical Chemistry, Faculty of Pharmacy, Medical University of Warsaw, Banacha 1, 02-093 Warsaw, Poland;

s078210@student.wum.edu.pl (M.J.); lukasz.szeleszczuk@wum.edu.pl (Ł.S.)

* Correspondence: dariusz.pisklak@wum.edu.pl

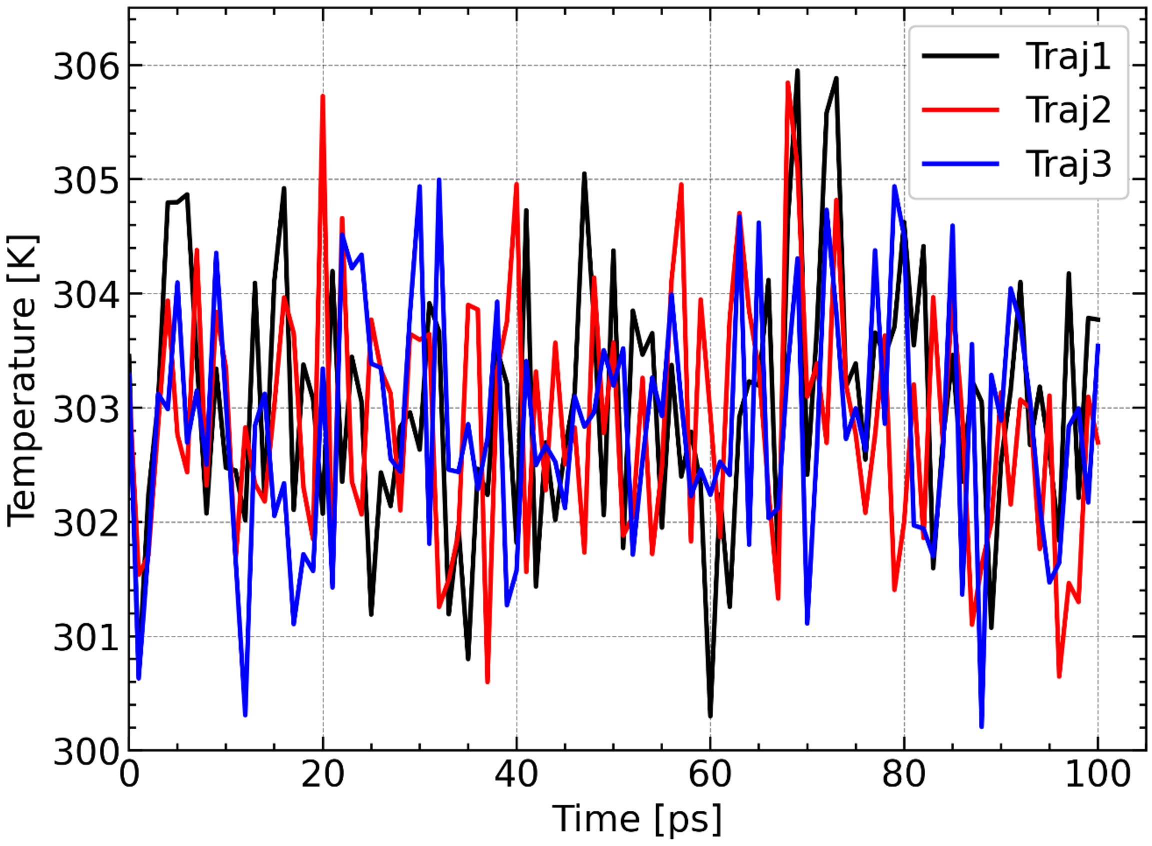

Figure S1. System temperature changes during NVT simulation for 3 different trajectories (Traj1, Traj2, Traj3).

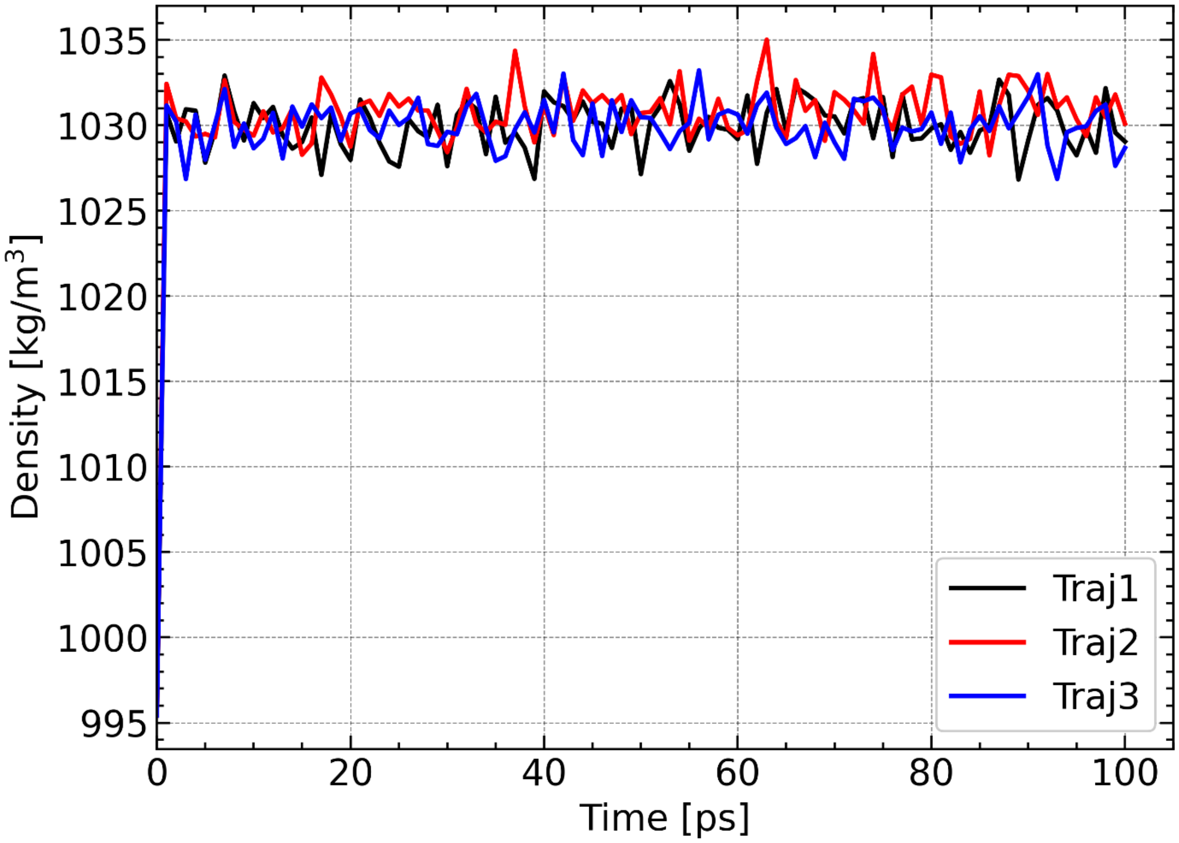

Figure S2. Changes in system density during NPT simulation for 3 different trajectories (Traj1, Traj2, Traj3).

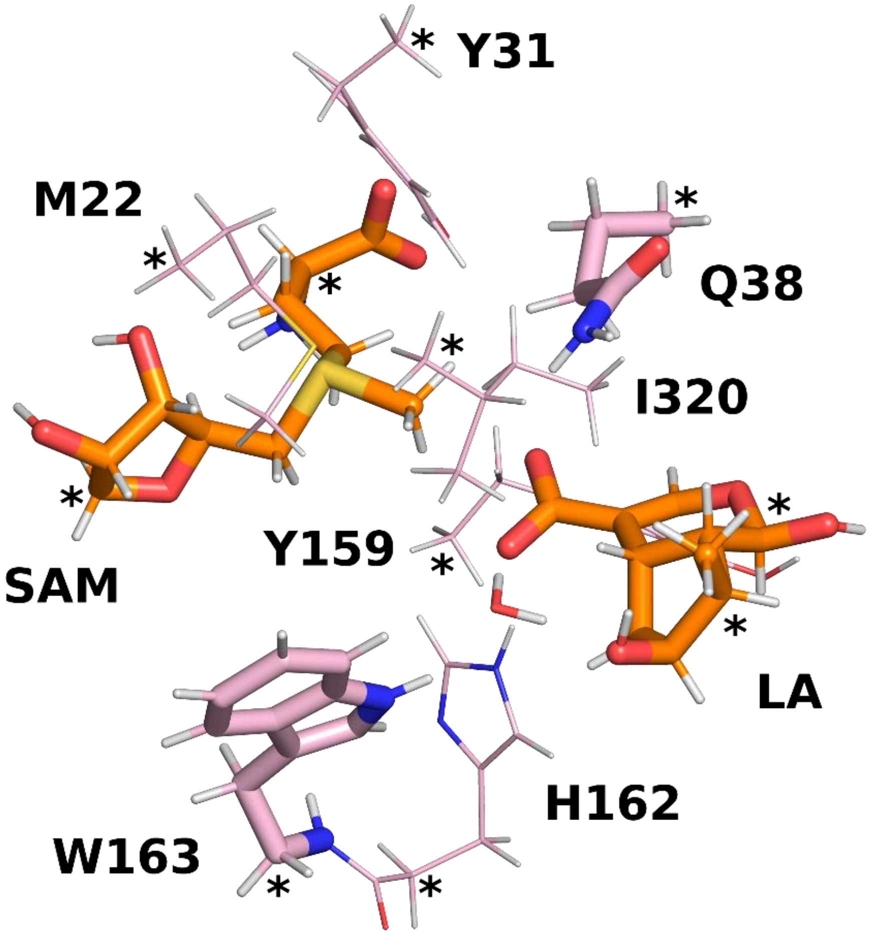

Figure S3. The structure of **Model 1** with water-mediated hydrogen bond between W163 and loganic acid. The fixed atoms are marked with an asterisk.

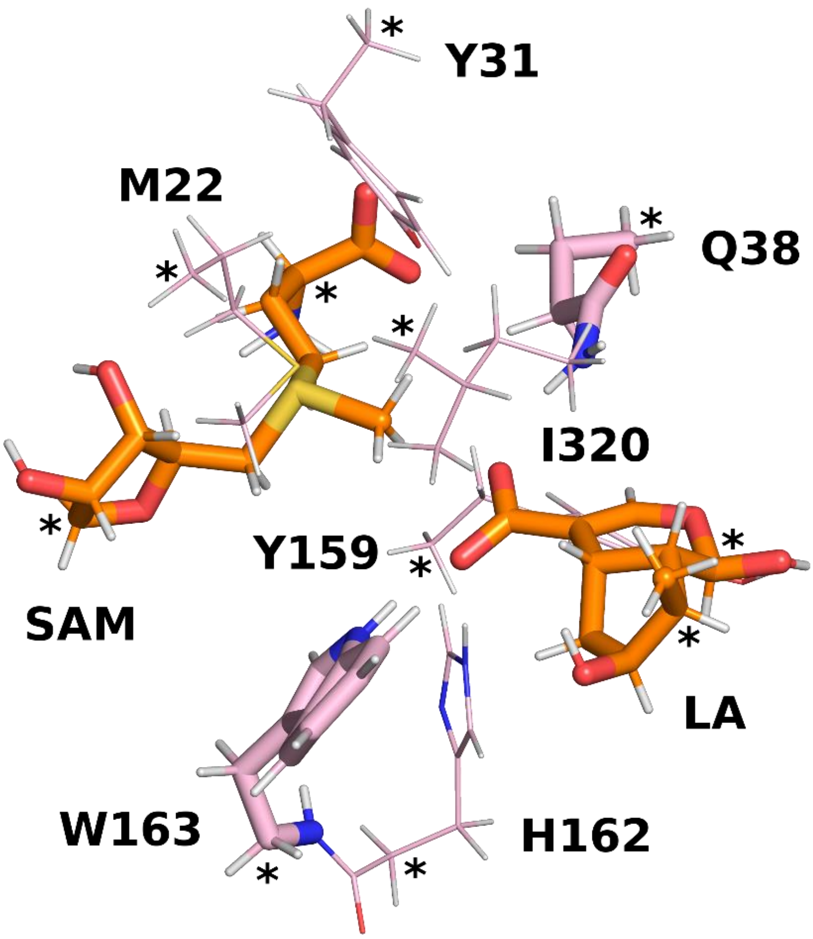

Figure S4. The structure of Model 2 with a direct hydrogen bond between W163 and loganic acid. The fixed atoms are indicated with an asterisk.

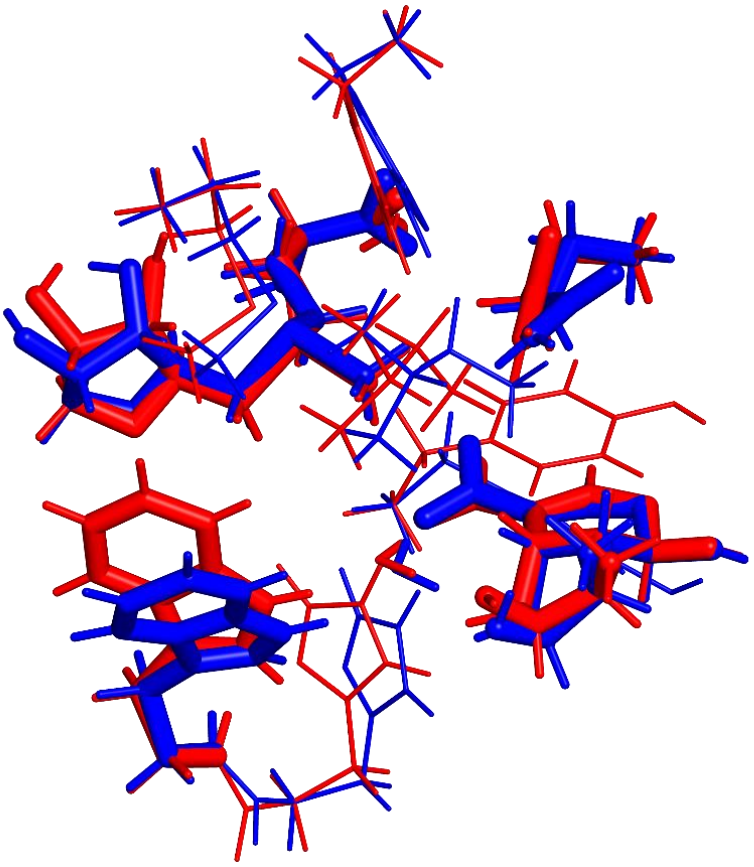

Figure S5. The initial structure from the optimization for Model 1 (in blue) superimposed on the structure of the found energy minimum (in red).

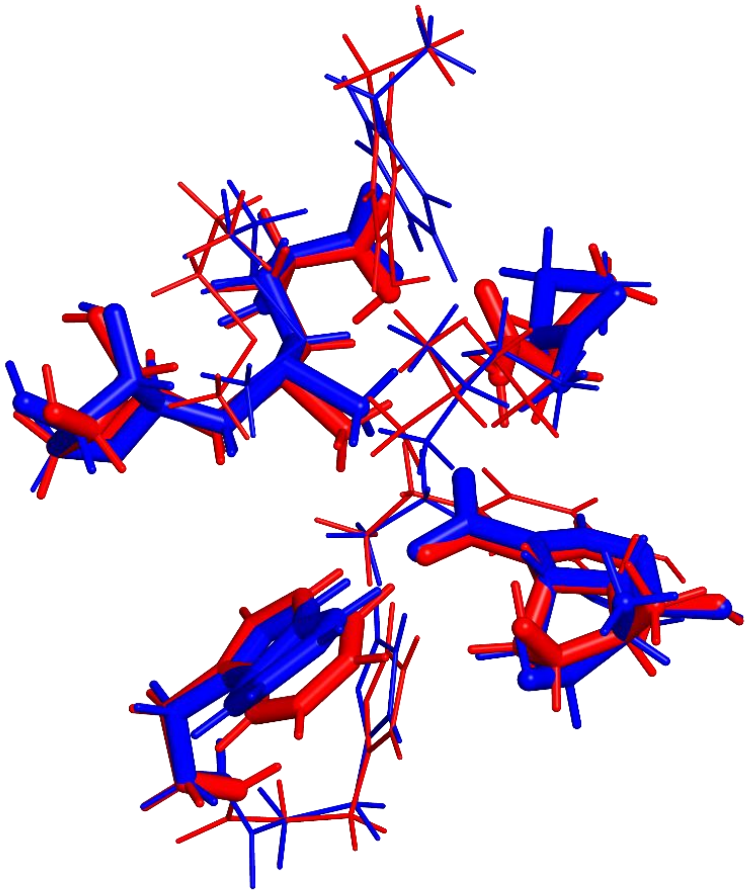

Figure S6. The initial structure from the optimization for Model 2 (in blue) superimposed on the structure of the found energy minimum (in red).

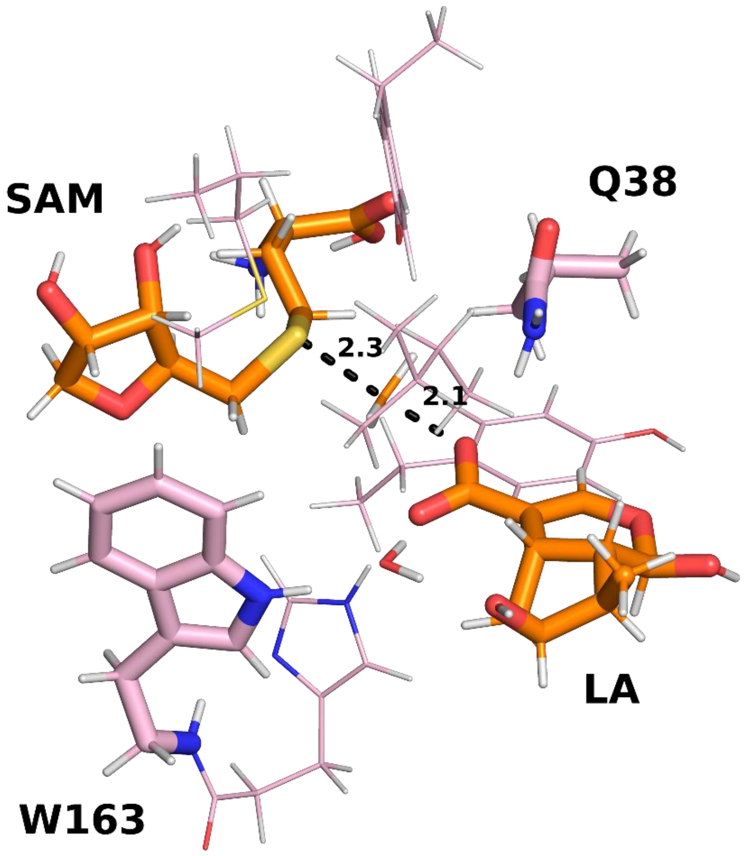

Figure S7. Optimized transition state structure for Model 1.

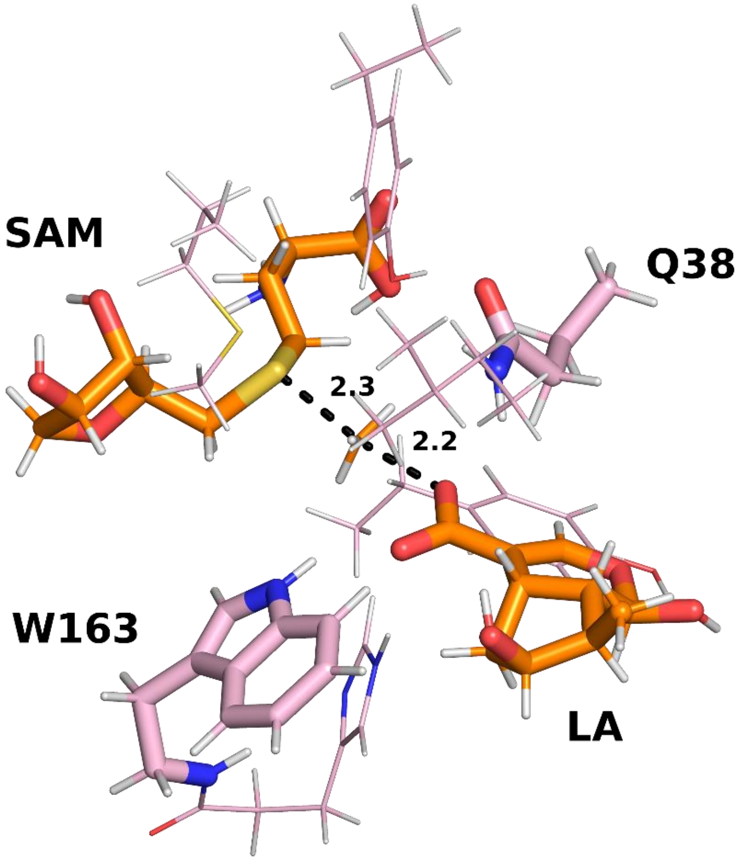

Figure S8. Optimized transition state structure for Model 2.
